# Recognizing Human Activity of Daily Living Using a Flexible Wearable for 3D Spine Pose Tracking

**DOI:** 10.3390/s23042066

**Published:** 2023-02-12

**Authors:** Mostafa Haghi, Arman Ershadi, Thomas M. Deserno

**Affiliations:** 1 Peter L. Reichertz Institute for Medical Informatics of TU Braunschweig and Hannover Medical School, 38106 Braunschweig, Lower Saxony, Germany; 2 Ubiquitous Computing Lab, Department of Computer Science, Konstanz University of Applied Sciences, 78462 Konstanz, Baden-Württemberg, Germany

**Keywords:** wearable device, neural network, human activity recognition, activity of daily living, quality of life

## Abstract

The World Health Organization recognizes physical activity as an influencing domain on quality of life. Monitoring, evaluating, and supervising it by wearable devices can contribute to the early detection and progress assessment of diseases such as Alzheimer’s, rehabilitation, and exercises in telehealth, as well as abrupt events such as a fall. In this work, we use a non-invasive and non-intrusive flexible wearable device for 3D spine pose measurement to monitor and classify physical activity. We develop a comprehensive protocol that consists of 10 indoor, 4 outdoor, and 8 transition states activities in three categories of static, dynamic, and transition in order to evaluate the applicability of the flexible wearable device in human activity recognition. We implement and compare the performance of three neural networks: long short-term memory (LSTM), convolutional neural network (CNN), and a hybrid model (CNN-LSTM). For ground truth, we use an accelerometer and strips data. LSTM reached an overall classification accuracy of 98% for all activities. The CNN model with accelerometer data delivered better performance in lying down (100%), static (standing = 82%, sitting = 75%), and dynamic (walking = 100%, running = 100%) positions. Data fusion improved the outputs in standing (92%) and sitting (94%), while LSTM with the strips data yielded a better performance in bending-related activities (bending forward = 49%, bending backward = 88%, bending right = 92%, and bending left = 100%), the combination of data fusion and principle components analysis further strengthened the output (bending forward = 100%, bending backward = 89%, bending right = 100%, and bending left = 100%). Moreover, the LSTM model detected the first transition state that is similar to fall with the accuracy of 84%. The results show that the wearable device can be used in a daily routine for activity monitoring, recognition, and exercise supervision, but still needs further improvement for fall detection.

## 1. Introduction

Alzheimer’s disease and other types of dementia are among the ten top non-communicable causes of death worldwide [1]. The World Health Organization (WHO) recognizes the domain of physical activities as one of six for quality of life (QoL). Tracking physical activities during the daily routine contributes to diagnostics and management of diseases such as Alzheimer’s, dementia, Parkinson’s, and depression [2,3,4]. Low back pain (LBP) is a common problem with many possible causes correlated with physical activity, including risky spinal postures and movements [5]. It can significantly limit daily activities. Monitoring the spine movement helps to understand LBP development as it indicates the activity of daily living (ADL) [6].

Middle-adulthood and elderly individuals have the highest prevalence rates of chronic LBP [7]. According to the United Nations (UN), the number of elderly above 60 years will exceed 16% of the world’s population by 2030 [8]. Despite the evident difficulties of living alone, 90% of the elderly prefer to live independently in their private spaces rather than in nursing homes and to maintain their independence, comfort, and privacy [9]. Recently, smart homes monitor parameters from several QoL domains such as (i) environmental, e.g., indoor air quality, (ii) behavioral, e.g., physical activity, (iii) physiological, e.g., vital signs, and (iv) psychological, e.g., emotion and stress. They utilize touch, interaction, and non-contact sensors to turn the home from a private space into a diagnostic space [10,11,12,13].

To this end, a key concern of elderly care in smart homes is the automatic evaluation of health-related parameters, in particular, ADL [14]. Thus, human activity recognition (HAR) during ADL needs to be developed. A variety of HAR implementations may enhance the QoL of the elderly. An efficient HAR system, for instance, ensures medication compliance, measures physical activities, and recognizes pathological conditions through continuous monitoring [15]. Wearable devices are recognized in home-based health monitoring for physiological measurements, gait analysis, and physical activity tracking [16]. Furthermore, they can track the posture of the lumbar spine whilst performing ADL [17].

The development of spinal movement analysis using the stationary devices has primarily revolved around the use of 3D motion tracking systems in a living labs setting. These systems create artificial environments for movement assessment, have limited capture volumes, are constrained by camera positioning, and provide complex descriptions of body segment movement. Hence, despite the thorough information that can be collected, these systems do not accurately represent real-world scenarios, are intrusive, violate privacy, and consequently, many of the elderly do not feel comfortable to live in the spaces in which such systems have been deployed [6,18].

In contrast, wearable devices are lightweight, compact, low-cost, energy efficient, portable, unobtrusive, and non-intrusive, which turns them into an appropriate choice for a broad range of indoor/outdoor applications from simple activity to more complex body segment kinematics estimation, particularly for lower limbs [19], gait analysis [20], and balance assessment [21]; additionally, it is also applicable for sleep evaluation [22], posture analysis [23], and fall detection [6,16,24].

Having said that, several approaches and wearables have been proposed for monitoring the physical activities using electrogoniometers [25], piezoresistive [26], microelectromechanical sensors (MEMSs) [27], strain gauge [28], accelerometers [29], inertial measurement units (IMUs) [30], force-sensitive resistors (FSRs), pressure sensors [31], and non wearables such as thermal infrared sensors [32], ultrasonic distance sensors [33], RGB and depth cameras [34], and capacitive sensors [35].

E. Papi et al. identified 22 wearable devices using technologies such as electrogoniometer, strain gauge, textile piezoresistive, and accelerometer assessing lumbar motion [6]. In addition, strain gauge is used by electrogoniometers to monitor changes in the angle between two end plates [36]. Epionics SPINE for example, deployed strain gauge in two sensor strips to evaluate the lumbar and thoraco-lumbar motions. Consmuller et al. found that Epionics SPINE is capable of assessing the lumbar spine shape and range of motion (RoM), having potential of evaluating LBP and rehabilitation [37]. Fragility, high expense, and complexity of instruction for end users in working with the sensor are the barriers to deploy the sensor in a daily routine. Bodyguard is a wearable device also deploying strain gauge technology which delivers a relative expression of the posture to the range of motion rather than absolute degree by computing spinal flexion/extension as a percentage of strain gauge elongation. O’Sullivan et al. investigated the performance of the sensor and found its potential as an effective tool for lumbopelvic posture monitoring in both clinical and laboratory settings [5]. Wong et al. introduced a system made up of three sensor modules, each having three uni-axial gyroscopes and one tri-axial accelerometer, along with the digital data acquisition and feedback system. These three sensor modules were located in the upper trunk, mid trunk, and pelvic level. Due to the distributed topology of the sensors, the device delivered an estimation of spinal curvature measurements [38].

In addition, there are several studies focusing on developing models and using artificial intelligence (AI) according to the available datasets. Shuvo et al. proposed a convolutional neural network (CNN) model that achieved a 97.71% accuracy on the UCI-HAR dataset. This dataset was recorded with a waist-mounted accelerometer and gyroscope sensor and consists of six activities, namely walking, walking upstairs, walking downstairs, sitting, standing, and lying [15]. Joshi et al. proposed a CNN-LSTM model and achieved an 86% accuracy on the WISDM dataset. The authors chose thirteen activities for this research, namely standing, jogging, stairs, sitting, walking, typing, writing, brushing, folding clothes, dribbling (basketball), playing (catch), eating (pasta), and clapping [39]. Perez-Gamboa et al. used the UCI-HAR dataset and suggested a CNN-LSTM hybrid model and achieved a 94.7% accuracy [40]. Wang et al. suggested a personalized recurrent neural network (RNN) or a variant of LSTM that reached a 96% accuracy on the WISDM (v2) [40]. However, there has not been an evaluated wearable device measuring comprehensive physical activities using both spinal movement and an accelerometer in both indoor and outdoor compliance and developing HAR during ADL. Our contributions in this work are as follows:

- Identifying an appropriate wearable device with multi-tasking capability coping with the daily routine of users;
- Designing and developing a comprehensive protocol in order to include a wide range of daily activities for evaluating the device functionality which contributes to rehabilitation, HAR, as well as fall detection;
- Developing, implementing, testing, and comparing three light neural networks in order to evaluate the functionality of the device;
- Dividing the study design into indoor, outdoor, and transition states activities, each with its own contributions and in a total of 22, i.e., ten indoor activities, four outdoor activities, and eight transition states;
- Evaluating the accelerometer, spinal, and fusion of data and identifying the correlated activities contributions.

The rest of this work is organized as follows: in Section 2 the materials, methods, and the study design is described. We present the results in Section 3. This is then followed by the discussion of the proposed approach and conclusion in Section 4.

## 2. Materials and Methods

### 2.1. Device Description

**Figure 1:**
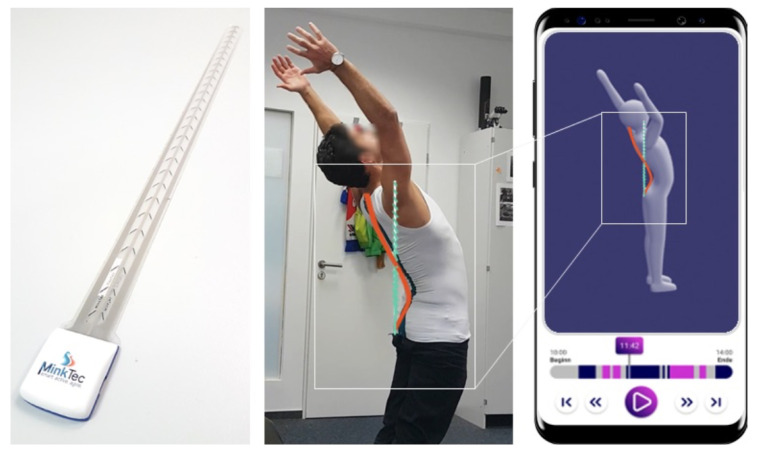
From left to right: Flexible wearable device, a subject wearing the wearable device, and the corresponding subject’s movements on a Minktec mobile application. The sensor’s strip can be bent to form a complete circle.

In this study, we used FlexTail® (Minktec, Braunschwieg, Lower Saxony, Germany), an up to 60 cm long and 2.5 cm wide sensor system consisting of an elongated strain gauge sensors strip and an electronics part at the bottom of the strip. There are 25 pairs of strain gauges on each side of the strip to detect 25 pairs of 3-dimensional measuring points every 2 cm in the longitudinal direction. Relative segment angles with a 0.5° precision per segment are recorded at an adjustable frequency, between 1 and 20 Hz. The electronics part contains a 3-axis accelerometer sensor, a lithium–ion battery, an internal memory, a microprocessor, as well as a Bluetooth and MicroUSB interface to transfer data to a smartphone or computer, respectively. The plastic casing dimensions of the electronics part are 4 cm × 4.5 cm. The device is placed on the spinal curve of the subject in order to log tilt changes in the spine. This is accomplished by having subjects wear a tight shirt provided by the company. The shirt has a longitudinal pocket on the back, holding the FlexTail firm but still allowing for a particular degree of freedom to slide longitudinally along the spine (Figure 1).

### 2.2. Study Design, Protocol, and Experiment

A total of thirty subjects were recruited to perform the experiment, of which twenty-two were males and eight were females, with an average age, weight, and height of 26.7, 73 kg, and 176.5 cm, respectively. All subjects were healthy (no known disease) and informed of the consent form. We designed the study comprising fourteen activities classified into three categories, namely static, dynamic, and transitions states (TSs), performed indoors or outdoors.

The study design and protocol is composed of ten indoor activities, four outdoor activities, and additional eight TSs. We recorded the indoor activities in the living lab, whilst asking the subject to go through the following protocol, sequentially:

(1) Standing, (2) sitting on the bed and lying down on right lateral decubitus (RLD), (3) turning, (4) and lying down on supine position, (5) turning, (6) and lying on left lateral decubitus (LLD), (7) changing the position from lying down to sitting and standing, (6) walking, (8) standing in the front of the armchair, (9) bending forward (BF), (10) back to the original position, (11) bending backwards, (BB) (12) back to the original position, (13) bending right (BR), (14) back to the original position, (15) bending left (BL), (16) back to the original position, (17) changing the status from standing to sitting, (18) sitting, (19) changing the position from sitting to standing, and end of the protocol. Therefore, the activities are composed of:

- Outdoor-performed dynamic activities; walking, jogging, running, and walking upstairs (WU) from the third floor to the fourth floor;
- Indoor-performed static activities; RLD, supine, LLD, walking, standing, BF, BB, BR, BL, and sitting. Of which nine out of ten activities, we categorize as the static activity (except walking, which is not associated with the movement but stand still);
- TSs; we considered the TSs as the performance between changing each two positions of static activities, if applicable. TSs include standing to lying down on the bed (TS1), rolling from RLD to supine (TS2), supine to LLD (TS3), standing to BF (TS4), BF to BB (TS5), BB to BR (TS6), BR to BL (TS7), and BL to sitting (TS8).

**Figure 2:**
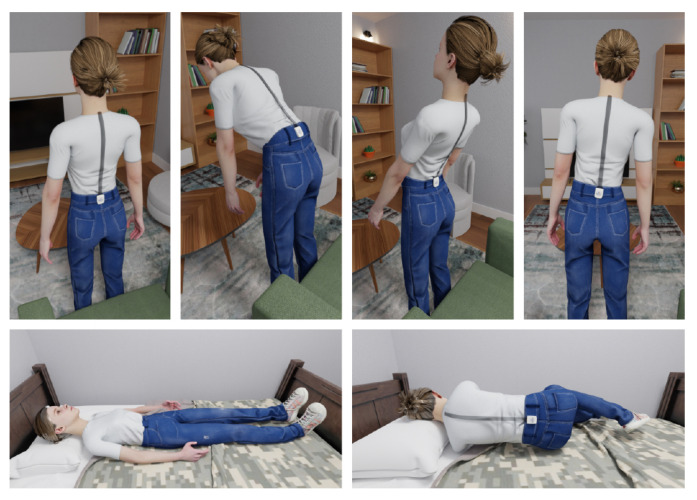
Top row from left to right: standing, BF, BB, and BR. Bottom row from left to right: supine and LLD positions. The study design included comprehensive physical activities contributing in wide-range of ADL.

Except for WU, each activity took one minute. For WU, each subject took as long as he/she needed to ascend the 23 stairs and one landing. Moreover, during BF, BB, BL, and BR, we asked the subjects to perform it up to their degree of comfort (Figure 2).

### 2.3. Data Acquisition

Although the device was capable of recording data at a frequency of up to 20 Hz, we have recorded the data in the sampling rate of 5 Hz, in order to secure the data acquisition and avoid data loss. After each subject completed all indoor and outdoor activities, the recorded data on the device in comma-separated values (CSV) format, which was transferred to the computer through MicroUSB. As an alternative, we also recorded the data over Bluetooth on a Raspberry 4B as the back up. Each CSV dataset contained 53 columns of data, of which, 50 columns the strain gauge sensor’s strip and three columns were the 3D accelerometer data.

### 2.4. Data Processing and Analysis

The recorded data of performed activities was labeled based on duration, accordingly. We developed two graphical user interfaces (GUIs) in order to reduce the risk of errors.

**Figure 3:**
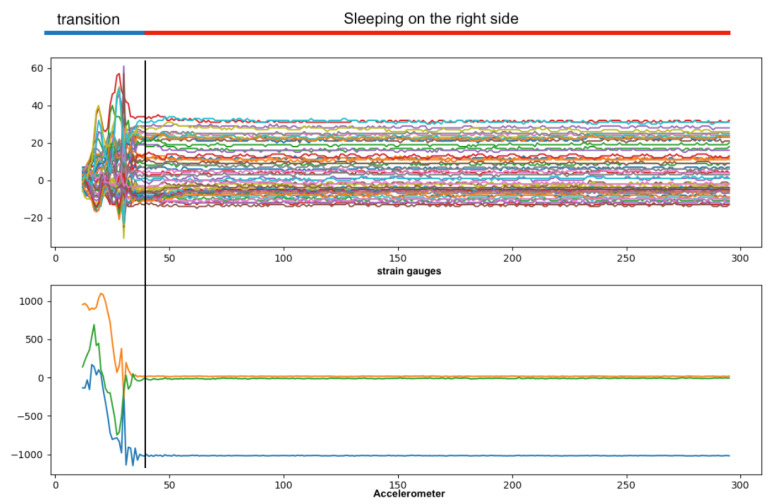
Labeling TS1 and RLD. We have labeled the TS activities based on the time period of previous activity and visual comparison.

**Figure 4:**
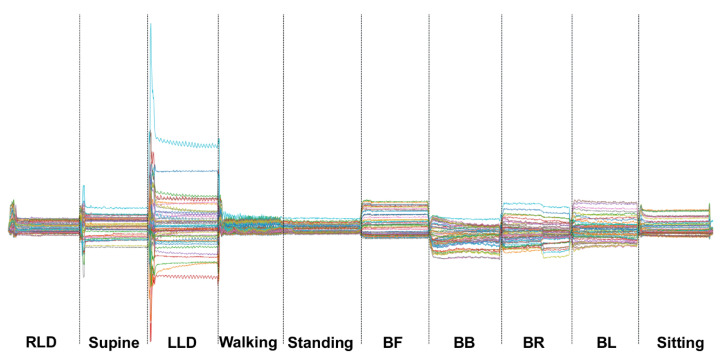
The recorded data for one of the subjects according to the protocol shows the indoor activities and data labeling based on time.

TSs were labeled based on the fixed duration of the previous activity and the visual amplitude differences between the transition segment’s signal and the other segments of the data (Figure 3 and Figure 4).

**Figure 5:**
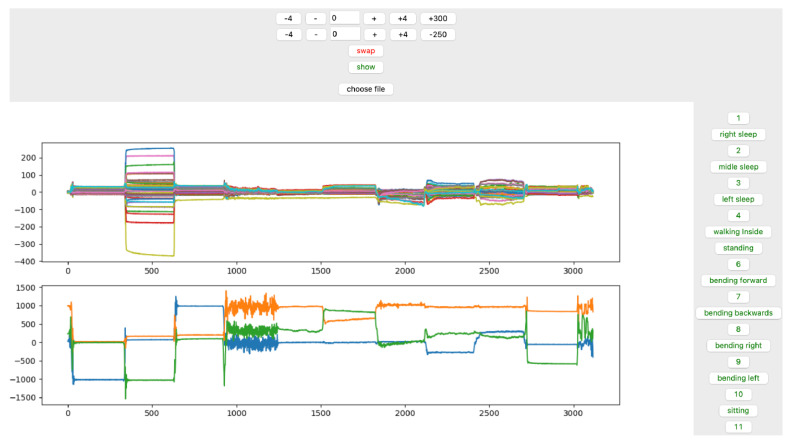
GUI for labeling indoor data. Time stamps and all three categories of activities are interactively configured and selected on the top and right side of the GUI, respectively; and in order to label the data, buttons on the right side of the panel can be used.

**Figure 6:**
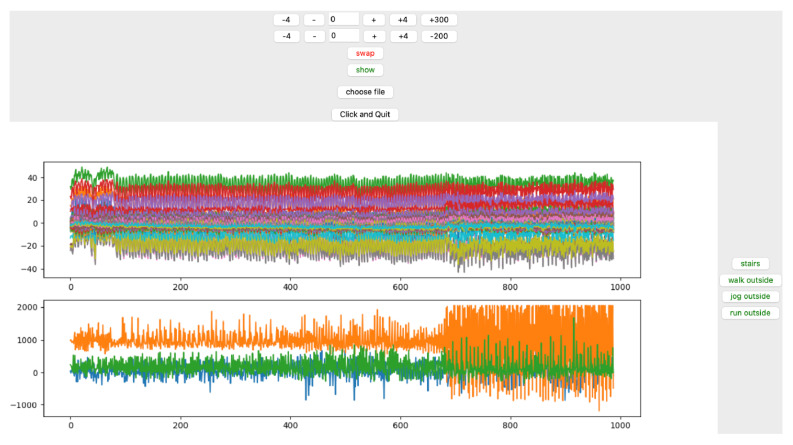
The GUI for outdoor activities. The initial experiment included four outdoor activities, which was reduced to three by combining walking and jogging in the later steps during data analysis.

The GUI is interactive in which the time stamp is set on top (Figure 5 and Figure 6). When configuring, the appropriate time stamp is performed by clicking on the corresponding button on the left side, and the associated data are labeled and stored in a separate CSV file.

Taking into account the physical activities and length of experiment, we have picked two window sizes of two and ten seconds with 50% overlap. In the case of ten-second window size, 3773 windows were created with 50-time steps and 53 features. As for the two-second window, 21266 windows were created with 10-time steps and the same number of features.

**Figure 7:**
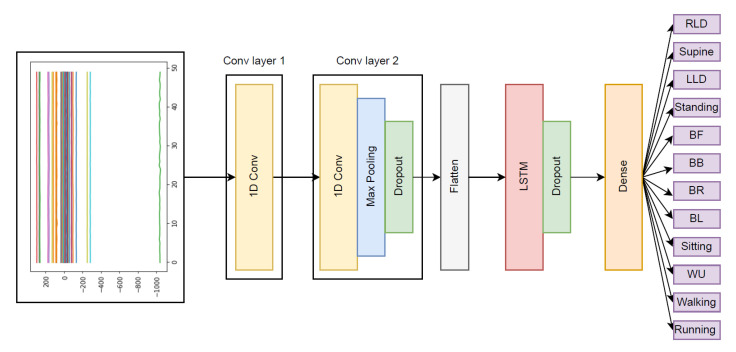
In addition to LSTM and CNN, we developed a hybrid model for activity classification.

In order to train and test the model, 77% and 23% of the windows were randomly picked. To develop the neural network and overcome the challenge of multivariate time series problem in activity classification, we developed the model based on the most suitable architecture for our work, i.e., long short-term memory (LSTM), convolutional neural network (CNN), and hybrid CNN-LSTM (Figure 7).

We trained the models according to two-second and ten-second windows. In each model, we have used two individual sets of data from accelerometer and strip sensors as well as five different states and combination units. The individual data and the units are:

- Accelerometer with three features;
- Sensor strip with 50 features;
- Fusion of sensor strip and accelerometer with 53 features;
- Sensor strip with dimensional reduced features using principle component analysis (PCA);
- Fusion of accelerometer with the PCA-generated features of sensor strip;

TSs were only included with the two-second windows, and data with the ten-second window were neither trained nor tested with TSs.

All the models were trained using Google Colab’s graphics processing units on account of the high number of epochs and the high number of features in the dataset.

## 3. Results

The study design and data acquisition were categorized into ten indoor activities, four outdoor activities, and eight TSs. During the data processing and analysis, we noted the similarity between two of the outdoor activities and inaccurate classification (i.e., walking and jogging). Therefore, we combined these activities into one category.

**Figure 8:**
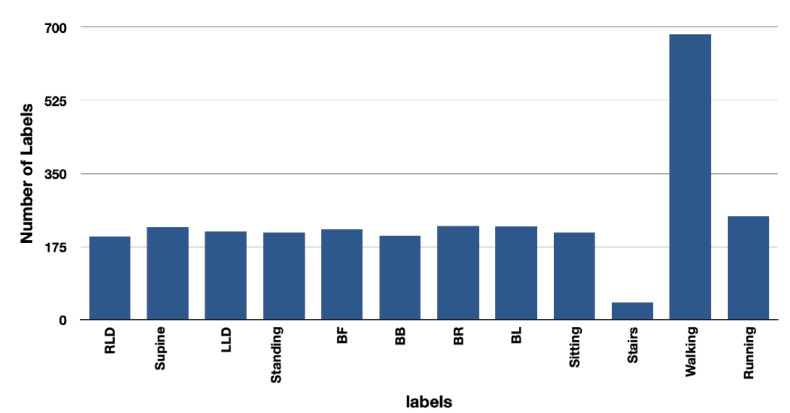
Number of labels for ten-second window is lower than for two-second window. Due to the nature of TSs, the models were not trained with this window size.

**Figure 9:**
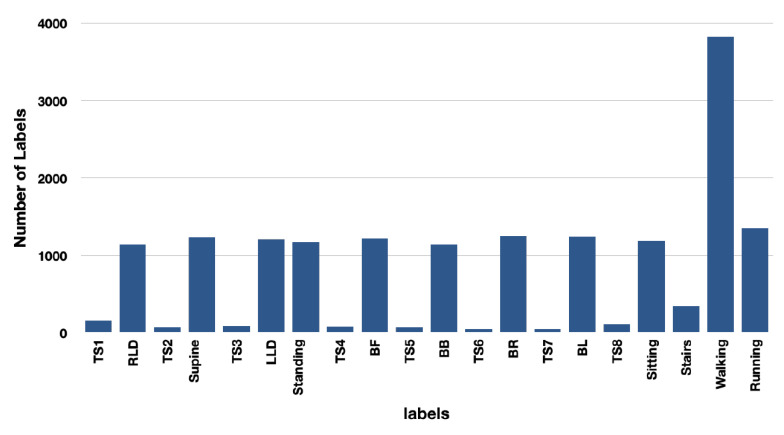
TSs are trained and tested with two-second window size. The number of labels for two-second window is significantly greater due to the combination of jogging and walking.

The distribution of windows created for each label using two and ten-second windows for the purpose of training the model is shown in Figure 8 and Figure 9.

We present the results of activities classification as follows:

### 3.1. Ten-Second window

#### 3.1.1. Architecture: CNN

**Figure 10:**
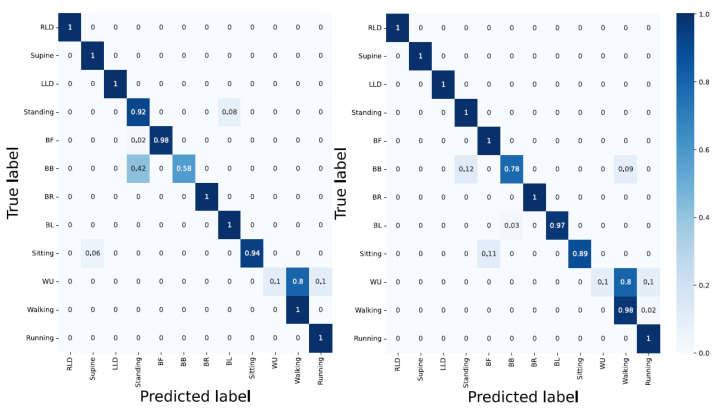
Performance of CNN network in activities classification. Left: without PCA applied and right: with PCA applied, which shows that the fusion of data significantly improve the network performance.

- Accelerometer; yielded an overall classification accuracy of 87%. Using only the accelerometer data resulted in a poor classification of at least four activities (standing = 82%, BB = 40%, Sitting = 75%, and WU = 0%). This is due to the similar characteristics of activities such as WU and walking.
- Sensors strip; gave the overall classification accuracy of 61%. On the one hand, activities such as BR, BL, and BB which are associated with spinal movements, delivered the best accuracy classification results with 100%, 97%, and 91%, respectively. On the other hand, static activities such as supine, LLD, standing, and RLD with 16%, 16%, 25%, and 30%, respectively, yielded the poorest results. This indicates the similar characteristics of spinal movements in static activities;
- Accelerometer and sensor strip; delivered better results compared with the individual data in unit one and two. The overall classification accuracy was 94%. However, WU and BB with 10% and 58% classification accuracy are still not correctly detected. This shows that convolutional layers could not differentiate WU from walking or BB with standing;
- PCA applied to the sensor strip; using 95% PCA, 50 features were reduced to 13. The overall classification accuracy slightly lowered, as CNNs need as many features as possible in order to perform pattern recognition;
- PCA applied to the sensors strip and accelerometer; with the PCA applied to the sensor strip and accelerometer data, the activities such as RLD, supine, LLD, standing, BF, BR, and running were 100% correctly detected (Figure 10).

#### 3.1.2. Architecture: LSTM

**Figure 11:**
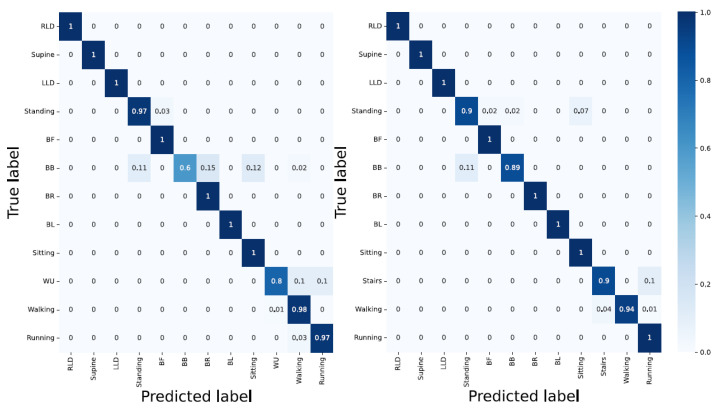
LSTM architecture with left and right, without and with PCA applied and data fusion. LSTM shows a promising performance in bending-related activities.

- Accelerometer; delivered an overall accuracy of 80%. RLD, supine, LLD, and running with 100% classification accuracy and WU, BB, and standing with 20%, 31%, and 43%, respectively, delivered the best and poorest results, respectively. Still, LSTM encountered difficulty in distinguishing the activities with similar patterns;
- Sensor strip; using sensors strip data feeding the LSTM model resulted in poor performance with an overall classification accuracy of 50%;
- Accelerometer and sensor strip; with 96% overall classification accuracy, the model classified RLD, supine, LLD, BF, BR, BL, and sitting without error. In addition, WU as one of the challenging activities reached an 80% accuracy. However, BB with a 60% classification accuracy remained a bottleneck. The reason for the false classification of WU can be laid on the landing step, in which the subject’s last step is considered as walking rather than ascending;
- PCA applied to the sensor strip; with 95% PCA, compared to sensor strip, better results were achieved. This can be due to the temporal dependency of LSTMs;
- PCA applied to the sensor strip and accelerometer; delivered 98% overall classification. A total of 9 out of 13 activities were correctly detected. For the other four, BB, standing, WU, and walking delivered 89%, 90%, 90%, and 94% classification accuracy, respectively, (Figure 11 and Table 1).

#### 3.1.3. Architecture: Hybrid CNN-LSTM

**Figure 12:**
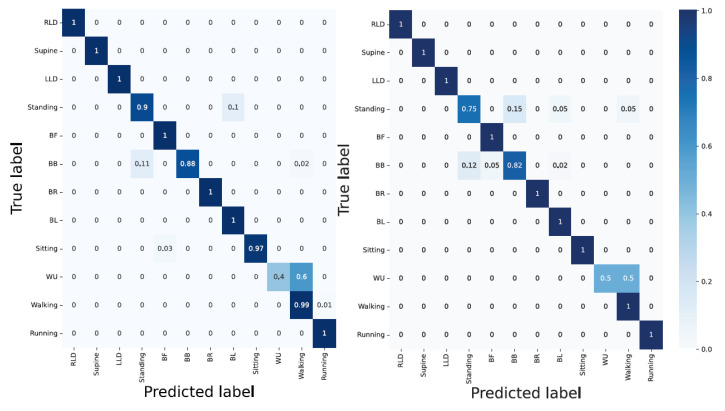
The hybrid model delivers a good performance with respect to lying down associated and bending activities, but is unable to resolve the WU challenge.

- Accelerometer; with an overall accuracy of 84%, the hybrid CNN-LSTM was unable to classify the activities such as standing, BB, WU, and sitting. This is inline with the result from the CNN;
- Sensor strip; delivered a very poor performance in classification. The results are similar to the CNN model;
- Accelerometer and sensor strip; yielded an overall classification accuracy of 97%. WU yielded an accuracy of 40% and RLD, supine, LLD, standing, BF, BR, and running were 100% correctly detected;
- PCA applied to the sensor strip; yielded better results compared with unit two. This showed that the LSTM layer compensates for fewer features;
- PCA applied to the sensor strip and accelerometer; the fusion improved classification in WU to 50%, and still supine, LLD, BF, BR, BL, sitting, walking, and running were detected correctly without fault (Figure 12 and Table 2).

### 3.2. Two-Second Window

#### 3.2.1. Architecture: CNN

**Figure 13:**
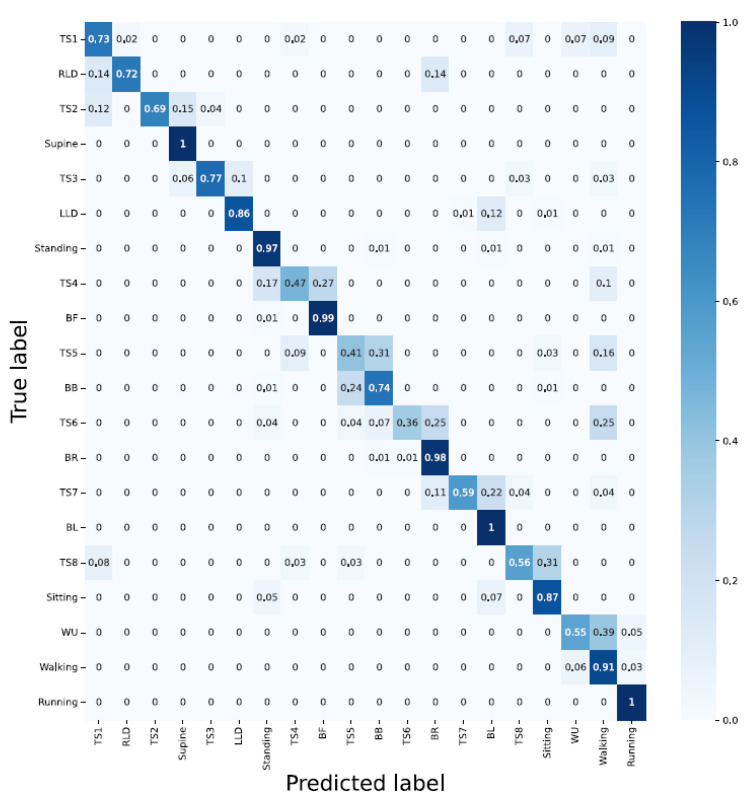
CNN architecture with the data fusion and PCA applied for two-second windows.

- Accelerometer; whilst the outdoor dynamic activities (walking = 99% and running = 97%), and lying down static position-associated activities (LLD, RLD, supine = 100%) delivered good results, the bending-related activities and the activities with similar characteristics and their corresponding TSs yielded a poor classification accuracy;
- Sensor strip; having compared the outcome of the CNN model using the sensor strip in two- and ten-second windows indicated the poor performance in all activities disregarding the static, dynamic, transition, indoor, and outdoor type of activities (except for BL). This indicates the unsuitability of the sensor strips data with the CNN model;
- Accelerometer and sensor strip; with the overall classification accuracy of 84%, the CNN model displayed a good performance for classifying both indoor and outdoor activities. However, the TSs delivered poor results (i.e., TS1 = 66%, TS2 = 69%, TS3 = 90%, TS4 = 40%, TS5 = 44%, TS6 = 57%, TS7 = 44%, and TS8 = 67%). Having less data for TSs explains the poor classification accuracy;
- PCA applied to the sensors strip; delivered poor performance. In particular, lying down-associated positions (RLD = 15%, supine = 23%, LLD = 30%), and static activities (standing = 38% and sitting = 59%), as well as all TSs yielded very poor results;
- PCA applied to the sensor strip and accelerometer; even though the TSs were still poorly detected, compared to unit three, the classification accuracy was improved by 1% (Figure 13).

#### 3.2.2. Architecture: LSTM

**Figure 14:**
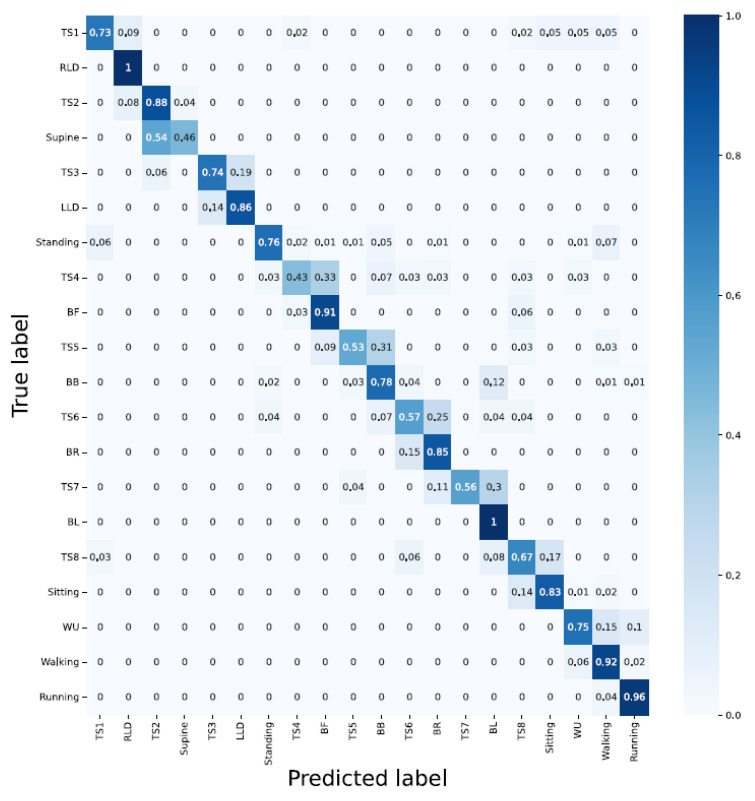
Using data fusion and PCA applied with LSTM architecture. Using two-second window downgrades the classification accuracy compared to ten-second windows, but TS1 and TS2 are detected with 73% and 88% classification accuracy, respectively.

- Accelerometer; except for running and walking and the dynamic outdoor activities delivering a classification accuracy greater than 90%, the remaining activities including TSs were poorly classified;
- Sensor strip; with 40% overall classification accuracy, RLD, and BB with 2% and 92% delivered the poorest and best outcome, respectively;
- Sensor strip and accelerometer; even though the data fusion improved the results, the TSs activities were inadequately detected. TSs were mainly wrongly classified into the previous and/or the next activity. This can be due to insufficient TSs data in comparison with other activities;
- PCA applied to the sensor strip; compared to unit 2, it seems that the overall classification accuracy was slightly improved;
- PCA applied to the sensor strip and accelerometer; RLD, BL, running, walking, and BF were detected with 100%, 100%, 96%, 92%, and 91% accuracy, respectively. TS4 and supine delivered the least classification accuracy with 43% and 46%, respectively (Figure 14).

#### 3.2.3. Architecture: Hybrid CNN-LSTM

**Figure 15:**
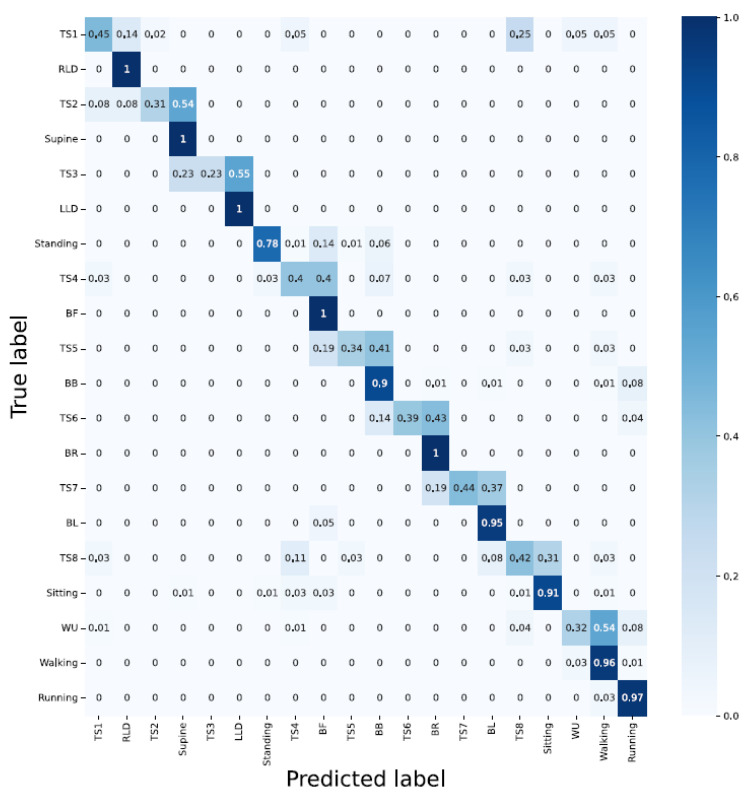
A hybrid architecture with two-second window delivers error-free results regarding lying down-associated positions activities and BR and BF.

- Accelerometer; in compliance with the previous architecture, the lying down-associated activities such as RLD, supine, and LLD delivered 100% classification accuracy while none of the TSs were detected with an accuracy greater than 50%. Moreover, the majority of bending-related activities were falsely classified. This is, again, similar to the CNN’s architecture, where bending-related activities were falsely recognized;
- Sensor strip; except for BL with a 98% classification accuracy, the architecture delivered a poor performance in almost all other activities;
- Sensor strip and accelerometer; data fusion improved the performance (RLD = 100%, supine = 100%, LLD = 100%, BF = 100%, BB = 98%, BR = 90%, BL = 100%, walking = 91%, and running = 98%). However, detecting TSs remained challenging;
- PCA applied to the sensor strip; although the general classification accuracy was low, it exhibited better results than unit 2;
- PCA applied to the sensor strip and accelerometer; results (i.e., TS1 = 45%, TS2 = 31%, TS3 = 23%, TS4 = 40%, TS5 = 34%, TS6 = 39%, TS7 = 44%, and TS8 = 42%) showed that correctly classifying the TS activities remained challenging (Figure 15 and Table 3).

## 4. Discussion

In this work, we deployed, tested, and verified the functionality of a multi-tasking, unobtrusive, and non-intrusive flexible wearable devices for simultaneously measuring and monitoring HAR and physical activities during ADL in various applications. By designing an extensive study protocol, we included several key physical activities which are frequently experienced by individuals during their daily routine.

In contrast with the previous works [5,37,38], the performance, applications, versatility, convenience, resolution, expense, and instruction in use have been significantly improved. Moreover, developing light and efficient algorithms made near real-time feedback to users possible. We also consider the solution as an in-home integrated evaluating system. Compared to [5,38], we have improved the mode of wearability and convenience of use for the users. The bulky set up, distributed hardware configuration, and complex user instruction in [5,40] were turned into a wireless data transmission, single sensor strip, and straightforward usage. The restricted applications (e.g., lumbo-pelvic posture and trunk movements) with limited activities (e.g., sitting, standing, and sitting activities) with a focus on sagittal and coronal planes have been extended to 22 various activities in three general states of indoor, outdoor, and TS activities. We have developed and applied the AI algorithms and tested the validity of the system in near real-time. In addition, the overall performance of the system in terms of classification accuracy has been improved to 98% compared to 86%, 94.7%, and 96% reported in [15,39,40], respectively. We have achieved this overall performance whilst reducing the window size, extending the number of activities, and improving the measuring resolution.

This study consisted of the activities which lead to pattern extraction, and contributes to physical activity-based diseases such as dementia, Parkinson’s, and depression whether for detection, prediction, or evaluation of the progress. In addition, the study was composed of activities which may contribute to therapy and rehabilitation in telemedicine.

Furthermore, we considered TSs as an important aspect of the study to investigate the contribution of the device in fall detection. This activity was simulated as the TS1 reflected standing to lying down with respect to the transition time and added value of accelerometer data. Moreover, it can contribute in assessing the correct performance of the therapy exercises.

We developed three light and efficient neural networks and evaluated the performance in a comprehensive manner of the broad range of activities of HAR recruiting 30 subjects. However, our work is restricted in terms of the number of subjects, and healthy subjects, and has not been tested with patients suffering from LBP nor in actual daily living. Alleviating the pandemic restrictions may give us the possibility to highlight this aspect in future studies.

By performing several experiments prior than the actual tests, we realized that the optimized sampling rate is 5 Hz. This was due to significant packet loss for the sampling rates greater than 5 Hz. Storing the data on internal memory would have enabled us to acquire the maximum rate (20 Hz). However, as we intended to transmit the data wirelessly and implement the algorithm on an embedded system in the future work, we preserved 5 Hz.

Due to the similar characteristics of the activities such as BB and BF as well as LLD, RLD, and supine, we expected similar classification accuracy. In some cases, BB and BF did not match. In particular, as for BB, we observed the inadequate sliding movement of the flexible wearable device in the shirt. As of evidence, we had to interrupt the experiment for two subjects, due to the misplacement of the device on the back of the subjects, whilst performing the activity supine to LLD. This was a challenge for the taller subjects rather than the shorter ones. The flexible wearable device had a rather good degree of freedom for sliding inside the shirt’s slot, having more than 10 cm of extra length. This extra length also did not contribute to spinal movement. In contrast, the taller subjects had the edge-to-edge of flex device and sliding slot, in which during the bending-related activities, the tip of the device could become stuck in the slot.

We relied on the activity duration and visual differentiation (for TSs) of signal in labeling. This could be further improved by using a synchronized camera. However, in addition to the increasing the complexity of this study, some of the subjects would have not agreed to be filmed.

In some cases such as walking, jogging, and running the collected data were the function of the subjects’ performance. The walking performance of one subject could be classified as the jogging performance of the other subject. This could be observed, in particular, for the comparison of men and women, leading to similar features and characteristics. Combining both jogging and walking in outdoor activities as one class of activity was the consequence of such an observation.

To implement a light and well-performing algorithm in activity classification. We tested the accelerometer and sensor strip individually. This drove us to find the added value of the data fusion of both data sources. We avoided increasing the complexity of the algorithm and load of data unnecessarily. This is of importance for implementing the algorithm on the embedded systems and micro controllers with limited computation power, in particular, for the applications demanding near real-time processing. We only used the most significant contributing features that yielded better or at least the same results compared with all 53 features.

From the implementation point of view on an embedded system, in the ten-second window, the CNN model with accelerometer data delivered the optimized classification accuracy for lying down-associated positions (RLD, supine, and LLD with 100%) as well as outdoor activities. In the CNN, the data fusion and joint PCA and data fusion improved the classification accuracy for the static activities including standing and sitting; standing = 82% and sitting = from 75% to 92%, 94% by data fusion, and to 89%, 98% by joint data fusion and PCA. LSTM yielded slightly better output for bending-related activities (BF = 49%, BB = 88%, BR = 92%, and BL = 100%). Moreover, WU as the most challenging activity reached the classification accuracy of 90% using LSTM architecture by joint PCA and fusion data.

In the bending-related activities, the hybrid architecture of LSTM-CNN suppressed the other two approaches. Applying data fusion and joint PCA improves the accuracy, even though there is no significant difference between fused data and joint PCA applied and fused data (6% in BB). However, this can lead to a large difference in practical implementation as the PCA has reduced the features from 50 to 13 in the sensor strip, and thus, the load of data, computational power, and data processing time are significantly lower.

The poor performance in the classification of WU is due to (i) a smaller load of data collected and (ii) the last step of the subject as a landing that can mislead the data to be categorized as a walking activity. This drawback can be resolved by increasing the number of subjects and an increment in the number of stairs.

We observed that the larger window size (ten-second) performed better in classification accuracy than the two-second window. However, still, the hybrid architecture with the joint PCA and fusion data performed well (RLD = 100, supine = 100, LLD = 100, standing = 78, sitting = 91, walking = 96, running = 97, BF = 100, BB = 90, BR = 100, and BL = 95).

Among all three neural networks for the ten-second window size, LSTM yielded the best classification accuracy. Even though the WU classification was a main challenge for the CNN and hybrid CNN-LSTM architecture due to the smaller sample size than other activities, LSTM performed adequately. Reducing the window size to two seconds and delivering the near real-time output, the hybrid CNN-LSTM architecture performed better. However, due to less samples, it yielded a poor classification of TSs.

The TSs, in particular TS1, could be of interest in smart homes, as they represent standing to laying down position, which could reflect a fall. Using LSTM with data fusion and joint data fusion and PCA, this was detected at 84% and 73% classification accuracy, respectively. We expect that training the model with further data could improve the TS1 classification accuracy.

To extend the application of the device, it can be used in verification of the physical activities in therapy and rehabilitation supervised by a medic, remotely, in order to observe whether the exercises have been performed correctly. Currently, such monitoring is carried out using cameras.

Monitoring the movement and analysis as well as improving the performance in sports such as Golf, which is directly associated with the back spinal, may be another application requiring further investigation.

As for the future work, the developed models can be implemented on a smartphone application and/or Raspberry pi 4B as an intermediate hub for real-time in-home monitoring of HAR.

## Figures and Tables

**Table 1:** While the lying down related, static, and outdoor activities are better classified using accelerometer data, the data fusion with the sensors strip and PCA application have the added value of improving the classification accuracy.

Architecture	RLD	Supine	LLD	Standing	Sitting	Walking	Running
CNN	100	100	100	82	75	100	100
LSTM	100	100	100	43	75	96	100
Hybrid	100	100	100	48	75	100	99
**Fusion**							
CNN	100	100	100	92	94	100	100
LSTM	100	100	100	97	100	98	97
Hybrid	100	100	100	90	97	99	100
**PCA and fusion**							
CNN	100	100	100	100	89	98	100
LSTM	100	100	100	90	100	94	100
Hybrid	100	100	100	75	100	100	100

**Table 2:** The individual data of sensors strip outperform the accelerometer data in the bending-related activities. The data fusion with the accelerometer further strengthens the performance.

Architecture	BF	BB	BR	BL
CNN	28	91	100	97
LSTM	49	88	92	100
Hybrid	45	98	62	99
**Fusion**				
CNN	98	58	100	100
LSTM	100	60	100	100
Hybrid	100	88	100	100
**PCA and fusion**				
CNN	100	78	100	97
LSTM	100	89	100	100
Hybrid	100	82	100	100

**Table 3:** Data fusion in LSTM architecture yielded a promising output for fall detection using the felx device.

Architecture	TS1	TS2	TS3	TS4	TS5	TS6	TS7	TS8
CNN	48	73	90	40	25	11	56	39
LSTM	65	85	87	30	31	29	48	47
Hybrid	48	46	42	10	0	14	26	44
**Fusion**								
CNN	66	69	90	40	44	57	47	67
LSTM	84	96	77	33	47	54	56	53
Hybrid	50	31	19	37	38	31	49	47
**PCA and fusion**								
CNN	73	69	77	47	41	36	59	56
LSTM	73	88	74	43	53	57	56	67
Hybrid	45	31	23	40	34	39	44	42

## Data Availability

Not applicable.
